# Hospital Finance

**Published:** 1922-11

**Authors:** 


### HOSPITAL FINANCE.

### THE PROGRESS OF THE COMBINED APPEAL.

THE Hospitals of London Combined Appeal seems in a fair way to achieve the collection of the hoped-for half million by the end of the year. As we go to press the total approaches £400,000, and the efforts of all concerned, and the generosity of the public, show no signs of slackening. Just before "Our Day and the Prince's Day," on October 6, Mr. William Ropner, of West Hartlepool, sent through the "Times" the handsome donation of £10,000. The organisation for the "Day" itself appeared to be very efficient, although it struck us that collectors would have done well to begin operations earlier at some of the London railway termini. Some 30,000 voluntary workers took part. Medical students, as usual, gathered in much money from an amused public. From Guy's 300 students with ten barrel-organs were out by 6 a.m. One of the organs was drawn by an "elephant," with a monkey mounted on its back. Two hundred University College students made up three concert parties, mounted on lorries, and a number of jazz bands. In the area covered by University College, success was largely due to the efforts of Sir Herbert Nield, who organised a wide appeal. Bart's contributed 250 students, and from the Middlesex Hospital 50 engaged in the work of collecting, a number of them dressed as sisters. Westminster Hospital provided 300 dental students to practise the art of extraction. The London Hospital organised a band of 300 workers for Stepney. Of the tales told by collectors we like best that of the street urchin who remarked, as a haughty person passed a flag-seller, head in air, "'E don't know what a horspital is; I 'ope 'e clicks for a haccident." A lady who called at an office in the Strand thought that her reception was not too cordial, but on leaving she was told, "We are not giving anything now, but to-night we are sending a cheque for £5,000." For the transport of collecting-boxes from Bart's to Cannon Street a taxi was chartered, and the driver refused to accept payment. Another taxi-driver gave his car and petrol free for the whole day.

Various local bodies in the London boroughs helped with pageants, carnivals and dances. Chief among these was the great display at Vauxhall Park, where the hospital procession arrived in the afternoon, and the Mayor of Lambeth opened a two days' fête. Canterbury Pilgrims, "Charlies," "Peelers," highwaymen, jazz bands, glee singers, niggers, dustmen and a mobile hospital ward appeared in the streets of Lambeth as collectors. In the pageant, "A Vision of Vauxhall," held in the evening, an attempt was made to revive the characteristic humours and excitements of Old Vauxhall. The prologue and lines, written by Mr. Claude Greening, were spoken by a native of Vauxhall, Mr. Wilkie Jones, the soloist being Miss Mabel Greatwood, the producer Mr. Henry Millar, and the conductor Mr. G. M. Sheen. The original bandstand with 2,000 lights was reproduced, and charming groups represented the changes of costume that have taken place during the long history of the gardens from about the year 1600 to the crinoline period.

A ball at the Hyde Park Hotel was attended by 400 guests. The programme included a Cabaret Follies entertainment, in which several well-known variety artists took part; tableaux depicting various phases of hospital life, dancing competitions, a tombola and a parade of mannequins.

The Prince of Wales had asked that the result should materially aid in carrying the aggregate result "into the last £100,000"—not, of course, that the last £100,000 should be raised in London on that day. When his message was published on September 15 the fund stood at £314,500; it has now reached £392,000. The exact figure for the Flag Day itself cannot be given separately. On October 18, the Organising Committee approved a recommendation of the Executive Committee in favour of an early distribution of £100,000, making a total distribution to date of £250,000.

The central organisation at work on the collection of the last £100,000 will have the use of Devonshire House during November and December, stands at the forthcoming Motor Show at Olympia and the White City (November 3 to 11), and a competition with £15,000 in prizes. Many civic committees and other helpers are organising balls during the winter, whilst the Amateur Players' Committee, under the chairmanship of Mr. Arthur Chapman and the secretaryship of Mr. Sydney Wallace, 28 Buckingham Palace Road, S.W.1, expects to hold over 100 performances in aid of the appeal. The various educational auxiliaries will also continue to give their help. Already 2,500,000 hospital stamps of one penny denomination and 250,000 sixpenny stamps have been issued. The total number of flags issued to October 6 was 600,000, and the number ordered for the School Flag Day in December is 1,500,000.

---

The late Sir James Ormiston Affleck, M.D., has left, in all, £8,000 to the Edinburgh Royal Infirmary, and £5,000 to the Edinburgh Royal Hospital for Incurables, among several other charitable bequests.

Mr. John Wells, of New York, who left £121,760, has bequeathed the residue of his estate to King Edward's Hospital Fund. On the death of his wife, he also leaves a sum amounting to £12,500 between four hospitals and an almshouse, the hospitals receiving the lion's share.

Its Finance Committee has advised the Manchester Corporation to distribute a sum of £6,000 to the hospitals of the city.

The residue of the estate of Mrs. Daniel Cork, of Cheltenham, amounting to £30,000, is bequeathed to eight London hospitals.

The Coventry Hospital Fête, including efforts ancillary to it, has produced £6,000 at the time of writing. A total of £10,000 is officially anticipated. The fête lasted for three days.

It is stated that a new and enlarged building is to be erected on the site of the old American Hospital in Paris, for which, in addition to sums in hand, a further £60,000 is required.

Representatives of the Carlisle Guardians and the medical profession have agreed in principle to a scheme for using part of Fusehill Poor Law Hospital for ten paying patients, at moderate fees. The average cost is expected to be £2 5s. a week.

The 30,000 people who lately crowded to view the new Princess Mary Infants' Ward at Leeds General Infirmary, voluntarily contributed £123 between them, or at the rate of less than one penny per head. The queues at times were a hundred yards long.

The residue of the estate of the late Mr. John Alcock, of Coventry, is understood to have been left to the Coventry and Warwickshire Hospital. An important benefaction is expected, and will be used to send to and maintain poor patients in convalescent homes.

The penny in the pound scheme at Sheffield has raised over £28,000, or £7,000 more than last year. An eventual total of £50,000 is expected, and it is hoped that Government and municipal departments may be empowered to act like other employers in the matter.

The governors of the Carnarvonshire and Anglesey Infirmary, Bangor, having failed to secure the workhouse infirmary, have decided to appoint a paid organiser to issue a public appeal, that the £ for £ grant offered by the British Red Cross Society may not be lost. This will mean the raising of at least another £4,000.

At a recent meeting of the Bradford City Council it was stated that St. Luke's Hospital, which was transferred two years ago as "a measure of economy" from the Guardians to the City Council, had cost £15,000 more to maintain than had been estimated.

The Haltwhistle and District War Memorial Hospital has been opened in Greencroft Park, Yorkshire. The building is a converted house which has been rented for 21 years. From funds in hand, when capital expenses have been met, an income of £250 is expected, and the town and district are invited to provide the balance required.

Mr. Wilfrid T. Allen, chairman of the Hospitals Welfare Society (S.E. London), has resigned. He has been invited to become president, in acknowledgment of his splendid services. Mr. Allen, who leaves the Society as "a friend and not as a carping critic," is succeeded by Mr. O. W. Hartley. It is hoped that Mr. Allen's example will be followed, and that no one will resign because he has left the chair.

In the area served by King's College Hospital already 121 firms, employing 4,000 persons, have adopted the weekly contribution scheme. At the recent rally on behalf of the hospital, Dr. Macnamara declared that if the scheme were not confined to industrial workers, it could embrace 150,000 people, which would mean £65,000 a year towards the £80,000 needed to maintain the closed wards.

Mr. F. N. Pickett, chairman of St. Paul's Hospital, Endell Street, has placed £15,000 in trust to found a research laboratory. Dr. David Thomson is honorary director, and Sir Ronald Ross, whose pupil he was, the president. It is hoped to endow a Ronald Ross research fellowship, and a yearly income of £1,500 has been raised by Dr. Thomson, Mr. E. R. Davies and Mr. E. G. Martens, Genatosan, Ltd.

Mr. H. Pike Pease, M.P., Assistant Postmaster-General, has written a pamphlet on the efforts of the Central Telegraph Office, St. Martin's-le-Grand, London, over their "Old English Bazaar," to be held in November, for the benefit of hospitals. The aim is to endow a bed for women at St. Bartholomew's Hospital.

At a meeting of Worcestershire Voluntary Hospitals Committee, in the Guest Hospital, Dudley, Mr. C. H. Baird, hon. secretary, reported that the Guest Hospital had received a grant of £840 from the Commissioners, having asked for £1,240; that the Worcester General Infirmary had been refused because it had shown "no new money"; that Kidderminster Hospital had been refused. One hospital delegate suggested that the smaller hospitals should be advised by the larger if they would accept such advice. This matter was left to the chairman. It was decided to ask the Commissioners if, should the present financial year remain unaltered, the grants would be affected.

Mr. E. W. Morris, house governor of the London Hospital, suggests an organisation for collecting produce, daily or twice a week, and sending it to the nearest hospital, to enable people who cannot help with money to send gifts in kind. Regularity of the service would be essential if the steward of the hospital were to dispense with purchasing supplies. At the London Hospital £10,500 a year is spent on milk, £4,000 on butter, £3,000 on vegetables and fruit, £3,000 on poultry and fish, and £2,400 on eggs. The hospital has already benefited by the conveyance of produce from a large garden near London, by a delivery company which did the work free of cost when it had empty vans returning to the metropolis.

### ALEXANDRA DAY.

At a meeting at the Mansion House of the Alexandra Day Distribution Committee, Miss C. May Beeman, the organiser, said that of the £45,000 raised in London after paying all expenses, including cost of flowers, the committee would be able to divide £37,587, or £6,300 more than last year. In the country £60,000 was raised; but the committee had nothing to do with the distribution of this. Since Alexandra Day was started 12 years ago more than £1,000,000 had been collected and distributed among hospitals, convalescent homes, and other institutions. This year £10,035 had been allocated to institutions in which Queen Alexandra took special interest. In distributing the remaining £27,000 the committee paid deference to the wishes of the mayors and local committees through whom the money had been collected.

